# Genetics of glutamate and its receptors in autism spectrum disorder

**DOI:** 10.1038/s41380-022-01506-w

**Published:** 2022-03-16

**Authors:** Sabah Nisar, Ajaz A. Bhat, Tariq Masoodi, Sheema Hashem, Sabah Akhtar, Tayyiba Akbar Ali, Sara Amjad, Sanjeev Chawla, Puneet Bagga, Michael P. Frenneaux, Ravinder Reddy, Khalid Fakhro, Mohammad Haris

**Affiliations:** 1 Laboratory of Molecular and Metabolic Imaging, Sidra Medicine, P.O. Box 26999 Doha, Qatar; 2 Shibli National College, Azamgarh, Uttar Pradesh 276001 India; 3 Department of Radiology, Perelman School of Medicine at the University of Pennsylvania, Philadelphia, PA 19104 USA; 4 Department of Diagnostic Imaging, St. Jude Children’s Research Hospital, Memphis, TN 38105 USA; 5 Academic Health System, Hamad Medical Corporation, P.O. Box 3050 Doha, Qatar; 6 Center for Advanced Metabolic Imaging in Precision Medicine, Department of Radiology, Perelman School of Medicine at the University of Pennsylvania, Philadelphia, PA 19104 USA; 7 Department of Human Genetics, Sidra Medicine, P.O. Box 26999 Doha, Qatar; 8 Department of Genetic Medicine, Weill Cornell Medical College, P.O. Box 24144 Doha, Qatar; 9 Laboratory of Animal Research, Qatar University, P.O. Box 2713 Doha, Qatar

**Keywords:** Neuroscience, Genetics

## Abstract

Autism spectrum disorder (ASD) is a neurodevelopmental impairment characterized by deficits in social interaction skills, impaired communication, and repetitive and restricted behaviors that are thought to be due to altered neurotransmission processes. The amino acid glutamate is an essential excitatory neurotransmitter in the human brain that regulates cognitive functions such as learning and memory, which are usually impaired in ASD. Over the last several years, increasing evidence from genetics, neuroimaging, protein expression, and animal model studies supporting the notion of altered glutamate metabolism has heightened the interest in evaluating glutamatergic dysfunction in ASD. Numerous pharmacological, behavioral, and imaging studies have demonstrated the imbalance in excitatory and inhibitory neurotransmitters, thus revealing the involvement of the glutamatergic system in ASD pathology. Here, we review the effects of genetic alterations on glutamate and its receptors in ASD and the role of non-invasive imaging modalities in detecting these changes. We also highlight the potential therapeutic targets associated with impaired glutamatergic pathways.

## Introduction

Autism spectrum disorder (ASD) comprises a broad range of conditions, including social, verbal, and repetitive behaviors with intellectual disability (ID). The cost of autism to society is increasing worldwide and is now $126 billion per year in the USA, more than three times the cost in 2006 [1]. Although the etiology of ASD is largely unknown, a broad scientific consensus points to genetics and environmental factors as predisposing characteristics in the development of autistic features.

Evidence from genetic and molecular studies delineates the impairment of synaptic function in ASD, with genes regulating synaptic functions being altered or mutated in ASD [2]. Individuals with ASD show alterations in brain development; however, the mechanism underlying the changes is unknown. The onset of ASD symptoms coincides with the timing of synapse formation and maturation, thus supporting the involvement of synaptic connections and neuronal function in ASD pathogenesis [3]. The arrested synaptic development in autism has been confirmed in human and animal studies, which found an abundance of thin, disrupted, and immature dendritic spines in different forms of ASD. Genes regulating synaptic structure and function are also highly mutated in ASD. The disrupted synaptic function in ASD relates to higher-level phenotypic changes as observed in other neurologic disorders and may result in altered sensory processing, cognitive deficits, hyperactivity, and seizures by affecting the balance between excitatory and inhibitory neurotransmission [4].

The abrupt synaptic connectivity relates to the alterations in glutamate receptor expression and function, subsequently modulating neuronal function [5]. The amino acid glutamate is the most abundant excitatory neurotransmitter in vertebrates and plays a significant role in neuronal development and cognition through its receptors. Defects in glutamate signaling are implicated in autism, but how such defects affect neuronal signal processing and cause varied autistic phenotypes remains unknown [6]. This review article describes how genetic changes affect glutamate and its receptors in ASD and highlights the role of non-invasive imaging modalities in detecting these changes.

### Glutamate receptors

Glutamate is an important excitatory neurotransmitter in the human brain. Glutamate receptors are implicated in various cognitive and neuronal developmental processes such as learning, memory formation, spine maturation, circuit development, and synaptic plasticity [7]. They are categorized as either ionotropic glutamate receptors (iGluRs) or metabotropic glutamate receptors (mGluRs).

The iGluRs are non-selective ion channels induced by glutamate and usher synaptic transmissions throughout the central nervous system [8]. These channels are subcategorized into N-methyl-d-aspartate (NMDA), kainate, 2-amino-3(3-hydroxy-5-methylisoxazol-4-yl) propionate (AMPA), or delta receptors based on their ligand-binding properties [9].

The mGluRs act through a second messenger and activate biochemical cascades that cause modification of ion channel proteins [10]. The eight types of mGluRs (mGluR1-mGluR8) are classified into three groups depending upon their structure and function. Group I mGluRs include mGluR1 and mGluR5, which are associated with the activation of phospholipase C [11]. Group II includes mGluR2 and mGluR3, and group III includes mGluR4 and mGluRs6-8 [11]. Altered glutamate signaling is associated with both syndromic and non-syndromic neurodevelopmental disorders.

### Mechanism of action of glutamate receptors

Glutamate released from the pre-synaptic membrane binds to iGluRs such as NMDA and AMPA receptors. All iGluRs are non-selective channels that facilitate the entry of cations such as Na^+^_,_ K^+^_,_ and sometimes Ca^2+^. The activation of NMDA, AMPA, and kainate receptors results in excitatory post-synaptic responses. In the NMDA receptor, an Mg^2+^ binding site binds Mg^2+^ in the presence of hyperpolarized membrane potentials and eventually blocks the opening of the NMDA receptor channel, thus restricting the entry of Ca^2+^ ions. However, when the synaptic membrane is depolarized, Mg^2+^ is removed, thereby allowing access to Ca^2+^ ions. The NMDA receptor enables the passing of Ca^2+^ ions only during depolarization of the post-synaptic cell, a property that is the basis for synaptic plasticity. Another interesting property of the NMDA receptor is that it requires co-agonists, such as glycine and glutamate, to occupy the binding sites for activation of the NMDA receptor.

**Fig. 1:**
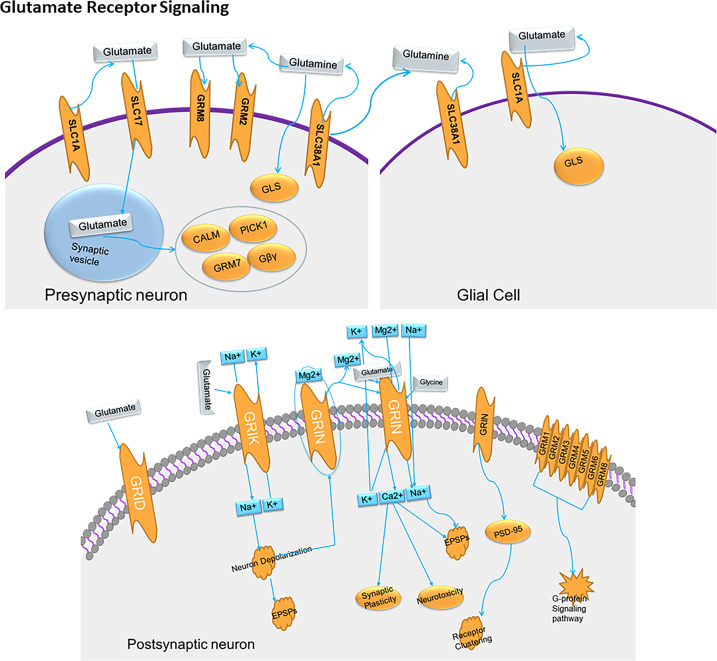
Glutamate signaling. Glutamate stored in pre-synaptic vesicles is removed for conversion into glutamine via reuptake by SLC1A into the pre-synaptic terminal or via uptake into glial cells. Glutamate re-enters pre-synaptic vesicles through SLC17A. Glutamine from glial cells has no neurotransmitter activity and is converted back to glutamate by GLS. The ionotropic glutamate receptors (iGluRs) (e.g., GRIN, GRIA, GRIK, and GRID) transport Na^+^ cations into the cell, resulting in Na^+^-mediated depolarization and development of excitatory post-synaptic potential (EPSP) in the post-synaptic membranes. Then, Ca_2_^+^ transport leads to the activation of Ca_2_^+^-dependent enzymes and ultimately to long-term post-synaptic modification. The mGluRs coupled with G proteins mediate intracellular signal transduction. GRM1 and GRM5 are related to changes in Ca_2_^+^ concentrations. GRM2, GRM3, GRM6, and GRM8 inhibit cAMP production. GRM7 prevents glutamate release from pre-synaptic vesicles.

Although both NMDA and AMPA receptors can allow entry for cations, weak stimulation causes the activation of only the AMPA receptor. In case of a strong stimulus, the AMPA receptor depolarizes the membrane sufficiently to expel the Mg^2+^ ion from the NMDA receptor and allow the entry of cations, including Ca^2+^. This results in a high intracellular concentration of Ca^2+^ that activates intracellular signaling cascades. In some cases, the intracellular Ca^2+^ binds to calmodulin (CAM), which further binds to the Ca²^+^/calmodulin-dependent protein kinase II (CAMKII) and phosphorylates the AMPA receptor on the synaptic membrane, resulting in increased Na^+^ conductance. CAM also promotes the movement of AMPA receptors from the intracellular stores to the synaptic membrane, thus creating more AMPA receptors to stimulate the post-synaptic neuron. As a result, the response to a given stimulus is more robust, leading to synaptic enhancement. This change is one of the mechanisms underlying long-term potentiation (LTP). However, mGluRs modulate post-synaptic channels indirectly and are sensitive to pharmacological agents. The binding of glutamate on mGluRs activates and dissociates G proteins, which then directly associate with ion channels or bind to other effector proteins such as enzymes (Fig. 1). Compared to iGluRs, mGluRs cause slower post-synaptic responses.

Kainate receptors are like AMPA receptors and allow the passing of cations, but they have limited distribution in the brain and exhibit a minor role in synaptic plasticity. Also, the selective activation of Gluk1 Kainate receptors elicits seizures in the basolateral amygdala region of wild-type and Gluk1 and Gluk2 knock-out mice [12].

### Metabotropic glutamate receptors and autism

Group I mGluRs comprising mGluR1 and mGluR5 are the most studied in autism and autism-related disorders. Altered functioning of the mGluR5 receptor occurs in Fragile X syndrome (FXS) [13], obsessive-compulsive disorder (OCD) [14], intellectual disability (ID) [15], and autism [16]. Higher levels of mGluR5 protein have been found in the vermis region of the cerebellum in children with autism than in unaffected children [17]. Similarly, a post-mortem study by Fatemi and Folsom (2011) found higher levels of the mGluR5 receptor in the superior frontal cortex of children with autism than in unaffected children [18]. The FXS is a major genetic cause of autism and is associated with the most common ASD phenotypes. Lohith et al. (2013) reported a higher expression of mGluR5 protein in the prefrontal cortex of patients with FXS than in healthy controls [19]. Fragile X mental retardation protein (FMRP), encoded by the *FMR1* gene, negatively regulates the synthesis of post-synaptic glutamate receptors and contributes to synaptic plasticity [20, 21]. A single gene mutation in FMRP leads to increased excitatory activity and altered synaptic function. Knock-out of *FMR1* results in mGluR-induced long-term depression (LTD) in animal models [22].

Genetic studies have revealed the presence of altered glutamatergic signaling pathways in ASD. Glutamate transporter genes are functional candidates for autism, and single-nucleotide polymorphisms (SNPs) in *SLC1A1* and *SLC1A2* glutamate transporter genes are associated with autism [23, 24]. The SNP rs301430 in the glutamate transporter gene *SLC1A1* is linked with repetitive behaviors and anxiety in children with ASD [25]. Moreover, Ramoz et al. (2004) reported that two SNPs (rs2056202 and rs2292813) in the mitochondrial aspartate/glutamate carrier gene *SLC25A12* are associated with autism [26].

Group I mGluRs are associated with NMDA receptors and therefore regulate the NMDA receptor-mediated LTP and LTD [27]. Studies using in situ hybridization and immunohistochemical analysis to assess the neuroanatomical localization of mGluRs demonstrated the presence of high mGluR1 expression levels in the olfactory bulb, cerebellum, thalamus, and hippocampus regions in the rodents brain [28]. However, mGluR5 was highly expressed in the forebrain and limbic structures [28]. Deleting or inhibiting the Group I mGluRs in rodents is associated with a reduction in learning and memory-associated tasks such as the Morris water maze task, which was the first evidence for the function of mGluR5 receptors in spatial learning using mGluR5 receptor knock-out animals [29]. In this task, mGluR5 receptor knock-out mice presented deficits in the acquisition and had impaired long-term retention [29]. In another study that used mice for the water maze task, inhibiting mGluR1 affected learning of new information but did not affect spatial information [30]. The use of the highly selective and strong mGluR5 receptor antagonist (3-[2-methyl-1,3-thiazol-4-yl)ethynyl]-pyridine (MPEP) reduced reference and working memory in a radial arm maze task in rats [31].

Transcriptomic analysis of the post-mortem brain of autistic individuals has shown that genes involved in synaptic function are downregulated [32, 33]. Many genes associated with ASD, such as neuroligin-3 (*NLGN3*), neuroligin-4 X linked (*NLGN4X*), neurexin1 (*NRXN1*), src homology-3 domain (SH3), and multiple Ankyrin repeat domains 3 (*SHANK*3) play a significant role in synaptic functioning [34].

Genomic studies using copy-number variation analysis have identified alterations in neurexins (pre-synaptic proteins) and neuroligins (post-synaptic proteins) in ASD [35]. Deletions of *NRXN* [36] or *NRXN3* [37] truncating mutations in *NRXN2* [38] and rare structural variations in *NRXN1* (*NRXN1*α and *NRXN1*β) are associated with the autism phenotype [39–41]. Neuroligins such as *NLGN1*, *NLGN2*, *NLGN3*, and *NLGN4* are implicated in ASD [42–45]. However, mutations of *NLGN3* and *NLGN4* are encountered in a small fraction of autism cases [46–49]. *NRXN1*α knock-out mice models show that α-neurexins are crucial for maintaining post-synaptic NMDA-receptor function, regulating synaptic transmission, and organizing the pre-synaptic terminals by coupling Ca^2+^ channels on the pre-synaptic surface [50–52], accompanied by behavioral abnormalities that resemble the core symptoms of ASD [53]. Similarly, mice with *NLGN3* mutations showed social and olfactory deficits [54], altered hippocampal synaptic plasticity [55, 56], impaired spatial learning [57], and altered synaptic transmission in the calyx of Held [44]. The alterations in neuronal circuits in autism may be reversible by re-expression of *NLGN3*, as an *NLGN3-*knockout mice model of non-syndromic autism showed impaired heterosynaptic competition and altered mGluR-dependent synaptic plasticity [58].

Group I mGluRs are implicated in *SHANK3*-dependent synaptic dysfunction in ASD [59]. *SHANK3* mutations impair mGluR-dependent LTD and attenuate the ability of hippocampal neurons to express group I metabotropic mGluRs at synapses [59]. Similarly, Heise et al. (2018) showed that compared to wild-type mice, SHANK3- and SHANK2-knockout mice had fewer glutamate receptors in the striatum and thalamus regions, while *CNTN4* knock-out mice had fewer glutamate receptors in the cortex and hippocampus regions [60]. Another gene, the G-protein coupled receptor-associated sorting protein-2 (*GPRASP2*), has been shown to play an important role in glutamatergic synapses. Deletion of *GPRASP2* causes impaired synaptic communications and modulates the surface availability of mGluR5 in mice [61]. *GPRASP* knock-out mice have abnormal mGluR signaling in the hippocampal neurons and exhibit ASD-like behaviors [61]. These results suggest that monogenetic variants in ASD-associated genes do not exhibit a common molecular phenotype excitatory and inhibitory signaling components, indicating that more research is required to explore the commonalities at different levels, such as information processing or neuronal activity networks in ASD [60].

### Ionotropic glutamate receptors and autism

Genetic alterations have also been linked to the NMDA class of iGluRs where both NMDA hyperfunction and hypofunction are associated with an ASD phenotype [62, 63]. However, an autoradiographic post-mortem study demonstrated no significant modifications in the expression of NMDA receptors in the hippocampal tissue from 4 males with autism [64]. Another post-mortem study found significantly increased NMDA receptor subunit 1 protein expression and reduced AMPA receptor density in the cerebellum tissue from 9 people with autism [65]. Disrupted NMDA signaling has also been implicated in a wide range of neuropsychiatric disorders other than ASD, including ID, schizophrenia, Alzheimer’s disease, and other mood disorders [66], suggesting different roles of NMDARs in synaptic plasticity and excitotoxicity [67, 68]. Moreover, causative mutations in *NMDAR* genes are implicated in ASD [69], and several ASD-related animal model studies have shown the association of ASD with NMDA abnormalities [70].

SHANK3, a post-synaptic protein at excitatory glutamatergic synapses that connects neurotransmitters and ions channels to the actin cytoskeleton, is implicated in ASD. Specifically, SHANK3 plays a pivotal role in regulating glutamatergic synapses, maturation of the dendritic spine, and strengthening the formation and transmission of synapses via glutamate receptors such as AMPARs and NMDARs. Therefore, mutations or deletions in *SHANK3* contribute to ASD symptoms [71]. The wide range of synaptic deficits observed in ASD-associated *SHANK3* rodent models are alterations in NMDARs [72]. Hence, alterations in NMDARs associated with the glutamatergic synapse are now identified as an important pathogenic pathway implicated in ASD.

Emerging evidence supports the role of iGluRs in synaptic plasticity, neuronal development, learning, memory, and cognitive processes. The expression and trafficking of iGluRs are regulated by neuronal post-synaptic density proteins, cytoskeletal proteins, and cell adhesion molecules [73]. A study identified a missense de novo mutation in the NMDAR subunit gene *GRIN2B* in a patient with ASD [74]. Furthermore, changes in AMPA and kainate receptors have been reported in ASD. A genetic study found that the interstitial deletion of chromosome 4q in a child with autism contributed to hemizygosity of the glutamate receptor AMPA2, glycine receptors GLRB and GLRA3, and neuropeptide receptors NPY1R and NPY5R [75]. Another study found that the glutamate receptor GluR6 was in linkage disequilibrium with ASD and suggested that chromosome 6q21 is a strong candidate region for autism [76]. In addition, a complex mutation in GluR6 co-segregates with non-syndromic autosomal recessive mental retardation, leading to the complete loss of Glu_k6_ protein and thereby to cognitive impairments [77].

In addition to *SHANK* genes, other genes such as *CNTN4* affect synapse formation and the excitatory and inhibitory signaling in ASD. Interestingly, the expression of glutamate receptors in the striatum and thalamus region of *Shank2-* and *Shank3αβ* knock-out mice is reduced. However, *Cntn4*-knockout mice show elevated levels of glutamate receptors in the striatum region and reduced levels of glutamate receptors in the hippocampus and cortex [60].

The cerebellum and hippocampus are two important regions that exhibit changes in iGluRs and are implicated in ASD. Mutations in neuronal synaptic proteins are critical in regulating the function of iGluRs. Mutations in neuronal synaptic proteins include post-synaptic proteins such as neuroligins (NLGNs) and pre-synaptic proteins such as neurexins (NRXN), SHANK, and GRIP1 [73]. As the number of post-mortem studies are limited, it is unclear whether these findings are true representations of regional expression variations between the hippocampus and cerebellum.

**Fig. 2:**
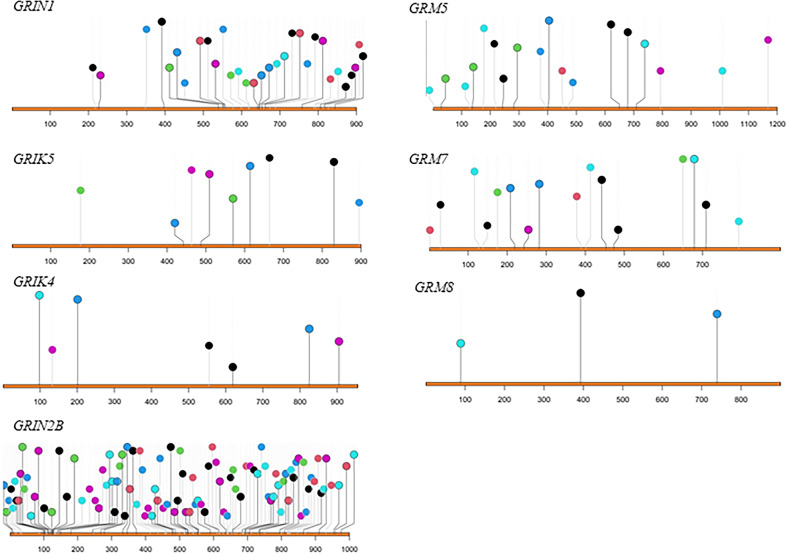
Distribution and amino acid location of mutations in ionotropic (GRIN1, GRIK5, GRIK4, GRIN2B) and metabotropic (GRM5, GRM7, GRM8) receptors. The data were downloaded using SFARI Gene database for autism spectrum disorder (https://gene.sfari.org/).

Several mutations have been observed in metabotropic and ionotropic receptors implicated in ASD susceptibility (Fig. 2). The majority of the mutations are observed in ionotropic receptors, with *GRIN2B, GRIN1*, and *GRM5* as the most susceptible receptors and *GRM8* and *GRIK4* as the least susceptible in ASD. Mutations are distributed evenly across the amino acids of both metabotropic and ionotropic receptors.

### Dynamic relationship of excitotoxicity, oxidative stress, and mitochondrial dysfunction

Recent studies [78, 79] have unraveled the role of oxidative stress in the pathophysiology of ASD. Children with ASD exhibit low plasma and cellular glutathione (an endogenous antioxidant) levels and reduced capacity of glutathione reserve; therefore, they are highly susceptible to oxidative stress. It has been documented that oxidative stress and redox imbalance are crucial components of ASD pathophysiology. Glutamate-mediated excitotoxicity has been reported as one of the essential contributing factors in developing oxidative stress in ASD [80]. Glutamic acid decarboxylase (GAD), an enzyme that catalyzes the transformation of glutamate to gamma-aminobutyric acid (GABA), glutamine synthase, and GABA receptors, are susceptible to oxidative injuries. The reduced levels of GAD in the brain promote excitotoxicity by decreasing GABA and increasing glutamate levels.

Accumulating evidence suggests that overstimulation of glutamate receptors, impaired mitochondrial functions, and oxidative stress are interconnected events that lead to oxidative neuronal injury in patients with autism [81, 82]. Typically, excitatory receptors allow the movement of sodium, calcium, and potassium, resulting in neuronal excitation. The movement of calcium into the cells results in the activation of inducible nitric oxide (iNOS) and phosphorylation of protein kinase C. Elevated levels of iNOS increase the production of free radicals, reactive oxygen, and nitrogen species, which start damaging lipids, proteins, and nucleic acids. Concurrently, protein kinase C induces phospholipase A2, which is involved in the production of pro-inflammatory molecules [83]. This cascade eventually generates free radicals that can initiate a wide range of toxic oxidative reactions, thereby potentially inhibiting oxidative phosphorylation and damaging mitochondrial enzymes that regulate the electron transport chain. Collectively, these events cause ATP depletion [84], eventually leading to energy deficits in neurons and cell death.

### Genes affecting neuronal migration, differentiation, and maturation in ASD

**Fig. 3:**
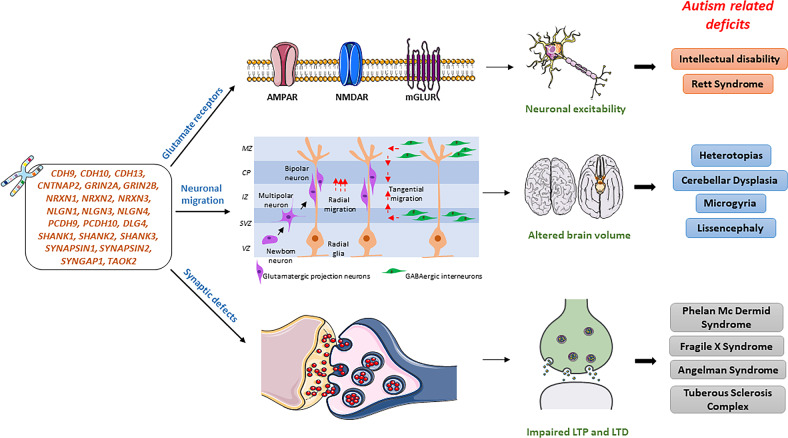
Genes optimal for normal functioning of glutamate receptors, neuronal migration, and synapsis. Mutations or disruptions in genes optimal for neuronal migration, synapsis, and normal functioning of glutamate receptors can cause neuronal excitability, altered brain volume, and impaired long-term LTP and LTD that lead to various autism-related deficits. MZ marginal zone, CP cortical plate, IZ intermediate zone, SVZ subventricular zone, VZ ventricular zone, LTP long term potentiation, LTD long term depression.

Modulation of neuronal activity is controlled by proteins that are involved in the pathophysiology of ASD. Genes that are optimal for the normal functioning of glutamate receptors and neuronal migration are found to be mutated or disrupted in ASD **(**Fig. 3**)**. Transcripts regulated by neuronal activity are present in high amounts in ASD candidate genes; for example, MEF2A/D, a transcription factor that plays a significant role in synapse development, regulates the genes *UBE3A*, *DIA1*, and *PCDH10*. The neuronal transcription factor NPAS4 plays an important role in the synaptic excitatory-inhibitory balance, and it’s expression is found to be regulated by neuronal activity. NPAS4 regulates the ASD candidate gene *NHE9*.

The expression of ASD candidate genes *UBE3B*, *CLTCL1*, *NCKAP5L*, and *ZNF18* is regulated by neuronal depolarization [85]. Mutations in a spliced variant of the *ANK2* gene known as Giant ankyrin-B (ankB) are involved in gene regulation and synaptic function and result in increased axonal branching and excitatory synapses during postnatal development [86]. *CNTNAP2* mutations in ectopic neurons also suggest their role in neuronal migration [87] and aberrant positioning of neurons in the corpus callosum. Moreover, the abnormal localization of CUX1-positive upper-layer neurons in the deeper layers has been reported in *CNTNAP2*-knockout mice, suggesting its function in core autistic deficits [88]. *CNTNAP2* also plays a critical role in radial-glia–driven neuronal movement as it encodes a neural transmembrane protein (contactin 2) that is involved in neural-glia interactions [89, 90]. A recent study reported that *CNTNAP2*-knockout mice exhibit decreased excitatory and inhibitory synaptic inputs onto the mPFC L2/3 pyramidal neurons, leading to aberrant neuronal firing in the cortical ensembles [91]. Another recent study demonstrated the significance of *NRXN-1α* in neuronal differentiation and the development of neural stem cells [92]. The study investigated the expression of *NRXN-1α* deletion during early neural induction in an autistic individual carrying a bi-allelic *NRXN-1α* deletion. The results showed that neural cells with the deletion had more radial-glia–like morphology and an increased number of differentiated astroglia [92]. Astrotactin 1 (*ASTN1*) and Astrotactin 2 (*ASTN2*) genes are neuronal cell surface antigens that are crucial for neuronal migration [93]. The interaction of *ASTN1* with *ASTN2* regulates neuron-glia adhesion [94]. The *ASTN2* gene has been implicated in ASD, and genome-wide association studies have shown that *ASTN2* is a candidate gene for ASD [95]. *ASTN2* deletions are associated with ASD and other neurodevelopmental complications such as attention deficit hyperactivity disorder, OCD, and language delay [96]. Moreover, the copy-number variations of *ASTN2* increase excitatory and inhibitory post-synaptic activity in individuals with ASD [97].

Distal-less homeobox (*DLX*) genes play a key role in neurodevelopmental disorders, including autism. They encode homeodomain transcription factors associated with the *Drosophila* distal-less (Dll) gene [98]. *DLX1* and *DLX2* genes are found in the transitory structure known as ganglionic eminences in the brain and regulate the migration of inhibitory interneurons from the medial ganglion eminence into the cortex [99]. There is a decrease in GABAergic neurons in *DLX1*-knockout mice, and the mice also exhibit epileptic behavior, which is a common pathology associated with ASD [100]. The downstream target of *DLX* is ARX, an X-linked homeobox gene that regulates the functioning of *DLX* in neuronal migration [101]. Patients with ARX mutations have autistic features [102].

*UBE3A* is highly expressed in the GABAergic and pyramidal neurons of the human cerebral cortex region. Deleting UBE3A in the GABAergic or inhibitory neurons of the Angelman syndrome (AS) mice model resulted in hyperexcitability and epileptic seizures, common abnormalities in ASD [103]. Additionally, a mutation of *rolled* MAPK3 at the 16p11.2 locus, an ASD mapped region, results in abnormal axonal targeting and fasciculation in *Drosophila* larval neuromuscular junctions [104]. *AUTS2* protein is mainly involved in gene expression regulation during brain development [105]. *AUTS2* is localized in the nucleus and in the cytoplasm, where it helps regulate neuronal migration and the growth of neurites [106]. Mice having mutations in *SHANK3*, a candidate gene for ASD, showed altered glutamatergic synapses in the pyramidal neurons of the ACC. Excitatory synaptic dysfunction and social deficits were observed in *SHANK3*-knockout mice, and the social behavior of the mice was improved by restoring *SHANK3* expression [107]. Moreover, *SHANK3* knock-out in mice demonstrated that *SHANK3* is associated with total brain volume and hippocampal size [108].

### In vivo evidence of increased expression of glutamate in autism

#### PET imaging

Non-invasive in vivo PET imaging is ideal for quantifying AMPARs and NMDARs in normal and diseased brains. PET tracers can be used to target NMDARs and AMPARs and can help facilitate both drug development and the pharmacokinetic profiling of candidate drugs [109]. PET probes are developed by targeting group 1 mGluR. These probes have already shown their specificity in binding with the Glu-5 receptor and have been implicated in studying Glu5R receptors in humans. The mGluR5 tracer [^18^F]-3-fluoro-5-[(pyridin-3-yl) ethynyl] benzonitrile ([^18^F]-FPEB) is used to measure the binding of mGluR5 in different brain regions. The binding potential of [^18^F]-FPEB was elevated in the cerebellum and postcentral gyrus regions of individuals with autism, suggesting altered mGluR5 binding in these regions [110].

A highly selective PET probe used for imaging the GluN2B subunit of the NMDA receptor is the benzazepine-based radioligand (*R*)*-* ^11^C-Me-NB. The efficacy of the PET probe has been demonstrated in rodents, which showed GluN2B-NMDA receptor expression in the cortex, striatum, thalamus, and hippocampus [111]. Using this probe, the NMDA receptor can be imaged non-invasively in autism, and the role of different genes on the expression of NMDA receptors can be evaluated in animal models. This PET probe can also be used to screen drugs targeting NMDA receptors. Several PET-based probes have been developed and tested to image iGluRs both in vitro and in vivo; however, none of them has yet been translated to clinical use, possibly due to their low binding affinity and impermeability to the blood-brain barrier. This provides an opportunity to develop a new class of PET ligands targeting iGluRs and potential clinical translatability. Nonetheless, these probes can be further developed to investigate the impact of gene mutations on glutamate receptors in animal models of autism.

### Magnetic resonance spectroscopy

Alterations in glutamate levels have been proposed to account for various behavioral and electrophysiological phenotypes in autism. The glutamate dysfunction is linked with abrupt neuronal function in autism. Alterations in the glutamate level in different brain areas may lead to different autistic phenotypes. Thus, quantifying the glutamate level in the brain of individuals with autism may provide valuable insights into the possible connections between altered genetic profiles and behavioral phenotypes of autism.

Proton magnetic resonance spectroscopy (^1^H MRS) is a well-established non-invasive approach used to ascertain the metabolic profile from different brain regions. The glutamate molecule consists of two methylene groups and a methine group that are firmly linked to form an AMNPQ spin system. Four protons present in two methylene groups form a prominent multiplet in the 2.04-2.35 ppm range on ^1^H MRS [112]. However, because of overlapping resonances with other metabolites such as glutamine, GABA, and NAA at lower field strength, their contributions are commonly combined and referred to as Glx.

Several ^1^H MRS studies have evaluated the alterations in Glx levels from different brain regions of individuals with autism. A ^1^H MRS study performed by Page et al. reported that the Glx concentration in the right hippocampus of 20 individuals with autism was higher than those of typically developing controls. Nonetheless, no significant changes were reported between the Glx level of the two groups in the right parietal cortex [113]. Although some studies have reported reduced Glx in the cerebellum [114], basal ganglia [115], and anterior cingulate cortex [116] in patients with ASD, others have reported elevated Glx levels in the anterior cingulate gyrus and auditory cortex regions in patients with ASD [117–119]. Glutamate levels that are significantly higher than those of controls have also been reported from the pregenual anterior cingulate cortex in pediatric patients with ASD, supporting the hypothesis that the activity of attention- and conflict-monitoring tasks are generally modulated in ASD [119]. Observations of higher or lower concentrations of Glx from different brain regions indicate that Glx levels are area-specific in ASD. However, the ratio of glutamate to glutamine in the Glx level remains unknown. Moreover, aberrations in glutamate and Glx levels in ASD differ between child and adult populations, suggesting that age is an important factor determining the excitatory or inhibitory functions in ASD.

Hassan and co-workers reported significantly higher blood and brain glutamate levels in children with autistic disorders [120]. In addition, the blood and brain glutamate levels were positively correlated, suggesting that blood glutamate levels can be utilized to diagnose autism early. ^1^H MRS analyses of single-gene disorders are strongly associated with ASD. Bruno and colleagues (2013) found reduced Glx in the caudate nucleus of patients with FXS [121], but Pan et al. (1999) reported a higher level of glutamate in the gray matter of girls with Rett syndrome [122]. The individual measurement of glutamate could provide a better idea about the synaptic alteration in autism. Using a higher field strength (7 T human scanner) provides better separation of peaks and allows precise quantification of glutamate levels in the brain [123, 124]. Another recent study by Horder et al. used ^1^H-MRS to measure the absolute glutamate concentration in the striatum and the medial prefrontal cortex in both humans and six rodent models of ASD [125]. They observed lower glutamate levels in the striatum of human ASD. Mice exposed to valproate showed decreased glutamate in the striatum, but BTBR T + tf/j mice showed higher glutamate in the striatum. Rats carrying *NLGN3* mutations showed significantly reduced glutamate both in the striatum and prefrontal cortex. This study emphasizes the varying effects of gene mutation on glutamate changes in the brain [125]. Individuals with ASD exhibited reduced functional connectivity and excitatory-to-inhibitory imbalance (glutamate + glutamine/GABA) in the cerebro-cerebellar region, with altered listening comprehension skills, suggesting altered glutamatergic signaling in the cerebellar regions of those with ASD [126].

Several studies have used single-voxel or single-slice multi‐voxel ^1^H MRS approaches to ascertain the metabolite pattern in patients with ASD but were constrained by limited spatial coverage. A substantial body of evidence [127] suggests that ASD is more diffuse than previously understood because more widespread brain dysfunctions have been reported from ASD patients. In contrast to single-voxel or single-slice ^1^H MRS sequences, three‐dimensional echo-planar spectroscopic imaging (3D EPSI) provides whole-brain metabolite maps with better spatial resolution [128, 129]. These volumetric maps can be spatially co‐registered to anatomical images to help in mapping metabolite variations from different brain areas with reduced possibility of partial volume averaging. Several studies have shown the potential of 3D EPSI in detecting metabolic abnormalities from multiple regions in patients with brain tumors [130, 131], neurodegenerative diseases [132–134], and neuropsychiatric disorders [135, 136]. We reason that 3D EPSI may be useful for detecting metabolic abnormalities, including glutamate, throughout the brain of patients with ASD.

Non-invasive detection of glutamate on conventional ^1^H MRS is challenging owing to increased resonance overlapping of glutamate with its neighboring metabolites such as glutamine, GABA, and NAA. However, two-dimensional correlated spectroscopy (2D COSY) resolves overlapping resonances of these metabolites unambiguously by introducing a second spectral dimension and identifying “cross-peak” resonances because of J-coupling interactions [137]. Moreover, an ultra-high-field (7 T) scanner further improves the sensitivity of 2D COSY in glutamate detection and other complex metabolites due to the high signal-to-noise ratio and increased chemical shift dispersion [138]. Due to its potential to isolate glutamate, glutamine, and GABA signals, 2D COSY may be an effective tool to investigate the impaired cortical excitation/inhibition equilibrium in ASD patients.

### GluCEST imaging

Chemical exchange saturation transfer (CEST) is a recently developed metabolic imaging technique used to detect low concentrations of specific endogenous and exogenous compounds. Studies have demonstrated that glutamate displays a concentration- and pH-dependent CEST effect at ~3.0 ppm downfield from bulk water protons [139]. Our group has established a glutamate imaging CEST approach (GluCEST) that can be used to create high-resolution parametric maps of glutamate changes in various neurological disorders. GluCEST detected decreased glutamate levels in the brain in a transgenic mouse model of Alzheimer’s disease [140]. In contrast, it showed a higher glutamate level in the brain in a mouse model of Parkinson’s disease [141].

Similarly, GluCEST detected altered brain glutamate concentration in patients with schizophrenia and temporal lobe epilepsy [142, 143]. In another study, GluCEST was used to monitor the modafinil-induced changes in glutamate levels in the rat brain [144]. Thus far, no GluCEST studies have been conducted in patients with autism, but this technique provides a novel approach to measure the level of glutamate changes in the brain. The GluCEST approach can be beneficial to determine the impact of a gene on the glutamate level in the brain. An animal model with different genes knocked out could be generated and imaged with GluCEST to understand better the effect of the gene mutation on regional alterations in the brain’s glutamate level. This method may explain how the mutation or deletion of particular genes links with regional brain glutamate level changes, thereby further enhancing our knowledge on the association of genes with specific neuronal functions.

### Treatments targeting the glutamatergic systems in autism

Substantive evidence suggests that inhibiting mGluRs can reverse autistic phenotypes in several ASD mouse models. Considering the substantial involvement of glutamate receptors in autism, different therapeutic strategies targeting these receptors are being developed to reduce ASD symptoms. Several drugs that target mGluRs are used to restore the excitation and inhibition balance in brain cortical regions. These include mGlu5 antagonists (fenobam), AMPA receptors (ampakines), and NMDA receptor inhibitors such as memantine, amantadine, and acamprosate [112, 145]. Glu5 antagonists improve the social and stereotypic behaviors in rodent models of ASD [146]. In the BTBR mouse model of autism, mGluR5 antagonist MPEP decreased repetitive self-grooming, whereas in three cohorts of BTBR mice, another antagonist of mGluR5, GRN-259, decreased repetitive behaviors. Moreover, both antagonists improved behavioral and communication skills in BTBR mice [147, 148]. Furthermore, treatment with AMPA compounds such as CX1837 and CX1739 reconditioned sociability in the sniffing parameter of the social approach task in BTBR mice [149].

**Fig. 4:**
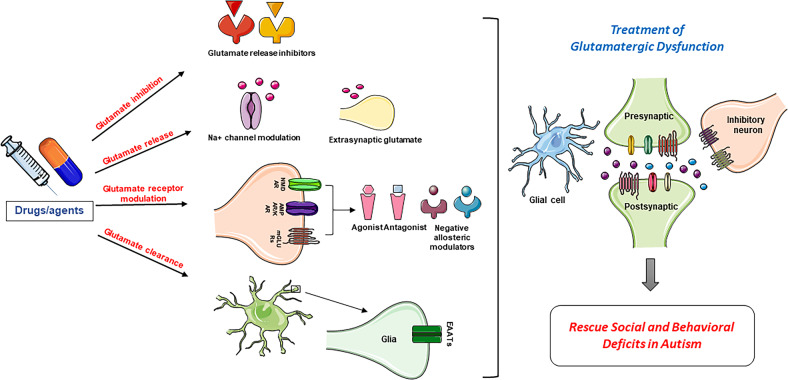
Potential therapeutic targets for developing drugs or agents targeting the glutamatergic system in autism. Different glutamatergic mechanisms such as glutamate inhibition, glutamate release, glutamate receptor modulation, and glutamate clearance serve as important targets that can be used for developing novel drugs or agents that can help in rescuing social and behavioral deficits in autism spectrum disorder.

Additionally, the non-competitive NMDA antagonist memantine has been utilized in clinical trials of ASD. Treatment with memantine reduced irritability, stereotypic behavior, and hyperactivity in children with ASD [150] and improved social behaviors in children with ASD [151, 152]. In addition, mGluR5 inhibition rescued autistic phenotypes in mouse models of ASD. One of the most common mutations in autism is the 16p11.2 microdeletion, and a study found altered mGluR5 synaptic plasticity in the hippocampus region of a mouse model of ASD with 16p11.2 deletion. These cognitive deficiencies in mice were reversed by treatment with a negative allosteric modulator of mGlur5 [153]. A study by Mehta et al. showed that the mGluR5 antagonist MPEP significantly decreased repetitive behaviors in valproic acid-treated mice [16]. An open-label clinical trial showed improved anxiety and pre-pulse inhibition in FXS individuals treated with the mGluR5 antagonist fenobam [154]. Single doses of mGluR1 antagonist (JNJ16259685) and fenobam rescued social and repetitive behaviors in eukaryotic initiation factor 4E-binding protein 2 gene (*Eif4ebp2*) knock-out mice [155], implying that group I mGluRs are a favorable therapeutic target for ASD. Results from these studies will permit researchers to gain better insights into the regulatory mechanisms of social behavior in ASD and help guide the development of novel AMPA receptor-based therapies to rectify social deficits implicated in ASD. In addition to glutamate receptor modulation by agonists, antagonists, and negative allosteric modulators, drugs or agents can be developed that can inhibit glutamate release, facilitate the clearance of glutamate by excitatory amino acid transporters, and modulate the voltage gated Na^+^ channels that are involved in glutamate regulation (Fig. 4). Subsequently, these modalities will be used for the treatment of glutamatergic dysfunction and would aid in rescuing social and behavioral deficits in ASD.

### Other mechanisms underlying ASD

Significant advances in delineating molecular mechanisms underlying ASD have provided insights into the neuroanatomical abnormalities implicated in ASD, such as enlargement of brain [156–161], hypoplasia of the cerebellar hemispheres, and vermis and decreased number of cerebellar Purkinje cells [162]. Studies on neural stem cells have demonstrated that abnormalities in neuronal morphogenesis, neurogenesis, synaptic function, and cell fate also contribute toward the development of ASD. In addition, Ca^2+^ signaling, chromatin changes, Wnt signaling, and RNA splicing also play an important role in the pathogenesis of these diseases. The neuropathological studies delineate the association between dysregulated fetal cortical development and ASD. The abnormalities associated with cortical development include increased neuronal number and decreased neuron size, misoriented pyramidal neurons, ectopic cells, dendritic abnormalities, irregular lamination, and reduced white matter tracts [163].

Several transcription factors are also involved in ASD due to their considerable effect on diverse neuronal functions. Transcription factors regulate the expression of many genes and their downstream target molecules. The X-linked gene methyl CpG binding protein 2 (MeCP2) is a transcription factor that represses gene function and influences downstream molecules implicated in ASD, such as brain-derived neurotrophic factor *(BDNF)* and *CDKL5* [164]. Engrailed-2 is another transcription factor that influences several biological processes implicated in ASD, as *Engrailed-2* is located in the ASD-susceptibility region on the human chromosome [165–167]. The involvement of Engrailed-2 in ASD is demonstrated by the finding that the Engrailed-2 null mice exhibited cognitive impairment and social dysfunction [168]. Moreover, Engrailed-2 is expressed upon activation of the Wnt signaling pathway; therefore, the Wnt pathway, along with Engrailed-2, also regulates axonal guidance and neuronal migration.

The Wnt pathway contributes significantly toward ASD because the genetic variants involved in ASD are increasingly found in genes of the WNT pathway [169]. In ASD, several *de novo* mutations are found in genes directly or indirectly related to the canonical Wnt pathway. These genes include mixed-lineage leukemia *(MLL)* complexes, members of the Brg1-associated factors *(BAF)*, *CHD8*, and T-box brain 1 *(TBR1)* [170–177]. Moreover, *Wnt2* genes found in the autism susceptibility chromosomal locus and several *Wnt2* variants are associated with ASD [178–182]. Correlation of the Wnt signaling pathway with ASD pathophysiology has also been demonstrated by the finding that the Wnt pathway is regulated by chromodomain-helicase-DNA-binding protein 8 (CHD8), and *de novo* mutations of *CHD8* are increasingly found in ASD individuals [183–185]. The modulation of the Wnt pathway in mice leads to the development of ASD-like social deficits and changes in the production of cortical neurons [186–189].

In addition to the Wnt pathway, the mTOR pathway has also been implicated in ASD. Mutations in the genes involved in the mTOR pathway affect the translation of neurons in ASD patients [190]. Studies in mice have demonstrated that dysregulation in mTOR signaling is associated with ASD behaviors [191–196]. The downstream signaling molecules of the mTOR pathway, such as 4EBP and eIF4E, are involved in ASD. Additionally, animal studies on mice demonstrated that the 4EBP2 knock-out and eIF4E overexpressed mice exhibited autistic-like behaviors [194]. The deficiency of phosphatase and tensin homolog (PTEN) hyperactivates the mTOR pathway, which contributes to altered social interaction and the development of macrocephaly [197].

Multiple regions of the brains of individuals with ASD contain activated astrocytes and microglia. The post-mortem studies on idiopathic ASD individuals have revealed that genes involved in activated astrocytes and microglia are upregulated in the cortex of the brain and to a lesser extent in the cerebellum [198, 199]. Although the genetic variations in the genes of astrocytes or microglia have not been reported, the knockdown of chemokine receptor 1 (*CX3CR1*) resulted in a reduction in microglia, ASD-associated functional connectivity, and behavioral deficits and defects in synaptic pruning [200, 201]. This indicated a potential association of this receptor to ASD. In addition, the upregulation of microglia and astrocytes causes dysregulated synaptic pruning, leading to synaptic dysfunction in ASD [202, 203].

Some neurotrophic factors, growth factors, and their receptors are also involved in ASD, such as MET receptor tyrosine kinase (RTK). The RTKs regulate several aspects of neuronal physiology, including neurogenesis and survival, differentiation and migration, patterned connectivity, and plasticity. The human gene *MET*, which encodes MET RTK, has emerged as a prominent risk factor for ASD. The mRNA and protein levels of MET are reduced in the cortex of ASD individuals [162, 204]. Therefore, failure in the transcription process of the MET protein is associated with ASD. In addition to MET, BDNF and the extracellular matrix glycoprotein Reelin are also associated with ASD [205–207]. The mutations in certain genes such as *PTEN*, fragile X mental retardation 1 (*FMR1*), chromodomain helicase DNA binding protein 7 (*CHD7*), and tuberous sclerosis 1 and 2 (*TSC1* and *TSC2*) are associated with the ASD [208–212].

Environmental factors and exposure to teratogens have been suggested as potential risk factors for ASD. The mother’s exposure to stress and valproic acid, thalidomide, or bacterial or viral infections can increase the risk of ASD in the offspring [213]. Valproic acid and thalidomide induce morphological abnormalities in the brain, such as reducing cranial motor neurons and modifying cerebellar structures [214, 215].

Thus, the complete understanding of ASD remains challenging due to a wide range of mechanisms underlying ASD pathophysiology. Identifying core mechanisms of ASD, such as the excitatory and inhibitory (E/I imbalance) mechanism involving the glutamatergic system, a common perturbation among ASD individuals, can help better understand the etiology of ASD. Moreover, the translation of human genetic and preclinical findings in ASD can provide mechanistic insights and clues to rescue different behavioral and synaptic deficits in early preclinical models, which may serve as a basis for future clinical trials.

## Conclusion

In the last few decades, investigations of genes and genetic loci have delineated the etiology of glutamatergic dysfunction in ASD. Moreover, the phenotypic penetrance of genetic variants overlaps with other disorders such as attention deficit hyperactivity disorder, which is highly dependent on an individual’s genetic background. Future studies should be focused on detecting low-penetrance variants and epistatic gene interactions and how these genetic factors are involved in the regulatory mechanism underlying the glutamatergic system in ASD. Furthermore, the glutamate receptors serve as a potential therapeutic target for ASD. Techniques such as MRS and PET imaging have been used to detect glutamate changes and to quantify NMDARs and AMPARs in the autistic brain, but the utilization of GluCEST imaging in patients with autism remains to be studied. The GluCEST approach can be instrumental in determining a gene’s effect on glutamate levels in the brain and can serve as a biomarker for detecting glutamatergic changes in ASD. In summary, glutamate has broad implications in the pathophysiology of autism, but more studies are required to explain how glutamatergic dysfunction drives core symptoms and deficits in ASD.
